# The Enhancement of Immunity Gained from Feline Trivalent Vaccines in Mice Using Feline IL-15, IL-23 and Metabolic Regulatory Molecules

**DOI:** 10.3390/biology14070834

**Published:** 2025-07-09

**Authors:** Ruichen Gao, Wei Sun, Danning Zhang, Linhan Zhang, Dafang He, Mengxi Li, Yi Wei, Junjie Peng, Gang Wang

**Affiliations:** 1 The Key Laboratory of Bio-Resource and Eco-Environment of Ministry of Education, College of Life Sciences, Sichuan University, Chengdu 610065, China; grc0130@163.com (R.G.); swei0427@163.com (W.S.); zdanning0407@163.com (D.Z.); inhanzhang@outlook.com (L.Z.); cxandhdfang@163.com (D.H.); limengxi0427@163.com (M.L.); 2 Chengdu Sanyoukang Biological Technology Company, Chengdu 610213, China; weiyi980807@126.com; 3 National Engineering Research Center for Biomaterial, Sichuan University, Chengdu 610065, China

**Keywords:** feline trivalent vaccines, immunity, adjuvant, IL-23, IL-15, metabolic modulators, Mn

## Abstract

The feline calicivirus, herpesvirus, and panleukopenia viruses are major infections that cause serious diseases in cats; however, current trivalent vaccines have limitations in immune efficacy and their duration of protection. This study assesses the immune-enhancing effects of novel adjuvants (feline IL-15, IL-23, and metabolic modulators) on vaccine responses. Forty mice were randomly assigned to four groups: Group A (composite adjuvants), Group B (metabolic regulatory molecules and Mn adjuvant), Group C1 (Mn adjuvant), and Group C2 (a blank commercial vaccine). The results showed that Group A had significantly higher neutralizing antibody titers against calicivirus post-booster immunization, while both Groups A and B exhibited enhanced antibody responses against the herpesvirus and panleukopenia viruses. Notably, Group A displayed increased proportions of memory T cells, follicular B cells, and activated B cells. These findings suggest that the combination of feline IL-15, IL-23, and metabolic modulators are safe and effective immunoadjuvants for trivalent feline vaccines to promote immune cell differentiation and antibody production, thus representing a promising strategy to optimize vaccine efficacy.

## Simple Summary

This study explores the immune-boosting effects of novel adjuvants—feline IL-15, IL-23, and metabolic modulators—on trivalent feline vaccines targeting calicivirus, herpesvirus, and panleukopenia viruses. Forty mice were divided into groups receiving composite adjuvants, metabolic molecules with manganese (Mn), Mn alone, or a commercial vaccine. The results indicate that the composite adjuvant group significantly elevated neutralizing antibody levels, especially post-booster, and enhanced memory T cells and activated B cells. The adjuvants were safe, with no adverse effects found on weight or hematology. These findings imply that IL-15, IL-23, and metabolic regulators can effectively enhance vaccine immunity, offering a promising approach to improving feline vaccine efficacy.

## 1. Introduction

Feline calicivirus disease, feline viral rhinotracheitis, and feline panleukopenia represent three prevalent infectious ailments in cats stemming from feline calicivirus (FCV), feline herpesvirus type 1 (FHV-1), and feline panleukopenia virus (FPV), respectively. FCV, categorized under the *Vesivirus* genus within the *Caliciviridae* family, is a single-stranded, positive-sense, non-enveloped RNA virus. Its high contagiousness can trigger sporadic outbreaks of upper respiratory tract diseases (URTDs) and virulent systemic diseases (FCV-VSDs) in cats, accompanied by a significant mortality rate [1]. FHV-1 is an enveloped dsDNA virus belonging to the *Varicellovirus* genus of the *Alphaherpesvirinae* subfamily in the *Herpesviridae* family and remains a primary pathogen causing upper respiratory tract infections. It spreads primarily via direct contact, contaminated objects, and droplets, leading to infectious rhinotracheitis in cats with high morbidity and mortality rates [2]. FPV is a small, non-enveloped single-stranded DNA virus belonging to the *Protoparvovirus* genus in the *Parvoviridae* family, which causes a common acute and often fatal illness in cats, characterized by severe diarrhea, vomiting, nasal discharge, and profound leukopenia [3].

The feline trivalent vaccine, officially titled the “Feline Panleukopenia, Rhinotracheitis, and Calicivirus Trivalent Vaccine,” is a multivalent formulation tailored for these three feline infectious diseases. It typically comes in inactivated or attenuated live forms. However, current practical applications of this vaccine exhibit certain limitations. The substantial antigenic variation among FCV strains limits the effectiveness of available vaccines against all strains, rendering suboptimal protection insufficient against infections by virulent wild strains or viral transmission among cats [4]. The current FHV-1 vaccine offers limited and short-lived protection, as the virus’s immune evasion tactics and multiple interferon response effectors often undermine vaccination efficacy [5,6]. Additionally, studies indicate potential immune evasion by FPV with FPV-251 demonstrating high virulence and immune evasion potential when confronted with serum from cats immunized with only the commercial vaccine in China (Feline Rhinotracheitis–Calicivirus–Panleukopenia Vaccine; inactivated virus) [7,8].

Adjuvants, comprising various molecules and materials, play a pivotal role in vaccine development by enhancing immune efficacy through prolonged antigen retention and sustained release, lymph node targeting, and dendritic cell activation modulation [9]. Manganese, a crucial trace element for immune regulation, among other physiological processes, is as a novel adjuvant candidate. Research shows that Mn^2+^ can bolster immune responses by activating the cGAS-STING and NLRP3 pathways, thereby enhancing antigen uptake, presentation, and germinal center formation [10,11]. However, the manganese adjuvant entails potential toxicity risks and inconsistent immune efficacy [11]. Traditional aluminum adjuvants, though widespread, often fail to elicit robust cellular immunity and may induce localized inflammation, making them less optimal for feline trivalent vaccines. Hence, safer and more effective immunological adjuvants are urgently needed in vaccine production.

Cytokines and metabolic regulatory molecules are indispensable for immune regulation. Cytokines like IL-15 exhibit multiple immunomodulatory activities, fostering the survival, proliferation, and activation of NK cells, B cells, and T cells [12]. IL-23 augments the immune response by promoting the expansion and survival of the Th17 subset of helper T cells [13]. Preliminary studies demonstrate that IL-15 and IL-23, when expressed as fusion proteins, significantly enhance mucosal immunity and systemic immune responses [14]. Numerous studies affirm that the nicotinamide adenine dinucleotide (NAD) and its precursors, β-nicotinamide mononucleotide (NMN), N-acetylcysteine (N-Ace), L-carnitine, sodium butyrate, and tryptophan, can impact immune efficacy by modulating immune cell activity and mitigating inflammatory responses [15,16,17,18,19,20,21,22].

To assess the adjuvant potential of these molecules, this study incorporated feline IL-15, IL-23, and a suite of metabolic regulators as a composite adjuvant, administered alongside trivalent vaccine antigens in mice. The aim was to evaluate whether this formulation could elevate vaccine immunogenicity and protective efficacy.

## 2. Materials and Methods

### 2.1. Mouse Grouping and Immunization Protocols

Forty female Kunming mice aged 6 weeks were randomly divided into four groups, with 10 mice in each group. Each group received subcutaneous dorsal immunization according to the protocol outlined in Table 1, followed by a booster for immunization at week 3. The Kunming mice were obtained from the Animal Experiment Center of Sichuan University (Chengdu, China).

The trivalent feline vaccine antigens used in this experiment (10^7.0^ TCID_50_ per dose of WH-2017 strain + 10^7.5^ TCID_50_ per dose of LZ-2016 strain + 10 ^5.5^ TCID_50_ per dose of CS-2016 strain) and the commercial vaccine “Maokangning” were provided by Sichuan Huapai Biotech (Group) Co., Ltd. (Chengdu, China). The inactivated FVRCP vaccine used in this study is a commercially available trivalent preparation comprising FHV-1, FCV, and FPV. Each viral component was propagated in CRFK cells and individually inactivated using beta-propiolactone (BPL) at a final concentration of 0.025% (*v*/*v*). The inactivation process was carried out at 4 °C for 24 h, followed by incubation at 37 °C for 2 h to allow for the complete hydrolysis of BPL. After inactivation, the viral components were mixed and formulated with an oil-based adjuvant to enhance immunogenicity. The vaccine was stored at 4 °C and used according to the manufacturer’s instructions.

### 2.2. Sample and Data Collection

#### 2.2.1. Body Weight Measurements

The mice in each group were weighed once a week for eight consecutive weeks to record the dynamic changes in the body weight of the mice in each group.

#### 2.2.2. Blood Immune Parameters

Whole blood was collected from mice via tail vein puncture using a sterile 1 mL syringe. For plasma preparation, blood was collected into tubes pre-coated with EDTA (anticoagulant), gently inverted several times to mix, and then centrifuged at 3000 rpm for 10 min at 4 °C. The supernatant (plasma) was carefully collected and stored at −80 °C until further use.

For serum preparation, blood was collected into plain, anticoagulant-free tubes and allowed to clot at room temperature for 30 min. The samples were then centrifuged at 3000 rpm for 10 min at 4 °C. The resulting sera were carefully collected and stored at −80 °C for analysis.

#### 2.2.3. Lymphocyte Subsets

On Day 56, after the first immunization, mice were euthanized, and their spleens were removed to prepare lymphocyte suspensions. The number of lymphocyte subsets was detected and analyzed.

### 2.3. Neutralizing/Antigen-Specific Antibody Detection

#### 2.3.1. The Detection of Feline Trivalent Neutralizing Antibody Levels Using the Fixed Virus Dilution of Sera

Anti-FCV neutralizing antibody levels were tested before the first immunization and on Days 7, 14, 28, 42, and 56 after the first immunization. Feline calicivirus (F9 strain) diluted to 100 TCID_50_ per unit dose was mixed with an equal volume of two-fold serially diluted test sera and incubated at 37 °C for 60 min. Crandell–Rees Feline Kidney cells are used for virus-neutralizing tests. Each dilution was inoculated into 3–6 wells of cells. After inoculation, the number of wells showing cytopathic effects (CPEs) in each group was recorded. The median protective dose (PD_50_) for each group was calculated using the Reed–Muench method, and the serum neutralizing titers were then determined [23].

Anti-FHV-1 neutralizing antibody levels were tested before the first immunization and on Days 7, 28, 42, and 56 after the first immunization. The sera, which were thermally inactivated at 56 °C for 30 min, were prepared with MEM in 96-well plates for serial two-fold dilutions. The diluted serum samples (50 μL) were incubated with an equal volume of FHV-1 (100 TCID_50_/50 μL) at 37 °C for 1 h, and F81 cells (100 μL) were added to each well (approximately 2 × 10^4^ cells); the plates were further incubated at 37 °C for 72 h. Neutralizing antibody titers were determined by observing the CPEs of the F81 cells. The LOD (limit of detection) represents the lowest detectable neutralizing antibody titer in the serum.

The fixed virus-diluted serum method was used to determine anti-FPV serum neutralizing antibodies before immunization and at 7, 28, 42, and 56 Days post-initial immunization. The FPV CS 2016 virus solution was diluted with DMEM culture medium to 100 TCID_50_/50 μL. An equal volume of serially two-fold-diluted test sera was mixed with the virus solution and incubated at 37 °C for 60 min. Each dilution was inoculated into well-grown F81 cells, with 3–6 wells per dilution for both the virus neutralization group and the virus control group. Normal cell controls were also included. The cultures were incubated at 37 °C with 5% CO_2_ for 96 h, and the number of wells showing CPEs was recorded. The 50% protective dose (PD_50_) was calculated using the Reed–Muench method, and the neutralizing antibody titer of the serum was then determined.

The antigen preparation was provided by Huapai Biotechnology (Group) Co., Ltd. (Chengdu, China) to ensure that the virus titer was consistent with that of commercial vaccines before fire extinguishing.

#### 2.3.2. In Vitro Detection of Feline Trivalent Antigen-Specific Antibodies

Specific antibody levels were tested in vitro before the first immunization and at 7, 28, 42, and 56 Days post-initial immunization. The levels of anti-FHV-1 antibodies (FHV-Ab) and anti-FPV-antibodies (FPV-Ab) in mouse plasma were measured in vitro according to the instructions for the Feline Trivalent Antibody Detection Kit (fluorescent immunochromatography, catalog number PRG108, Shanghai Glinx Biotechnology Co., Ltd., Shanghai, China) [24].

### 2.4. Immune Cell Count in Blood

Complete blood count (CBC) analysis was performed on blood samples from each group before the first immunization and at 7, 14, 28, 42, and 56 Days post-initial immunization. A 50 µL sample of whole blood containing the EDTA anticoagulant was taken from each group and analyzed using the veterinary fully automated five-part differential blood analyzer (TEK-VET5) manufactured by Tekon Biotechnology Co., Ltd. (Chengdu, China), according to the operating procedures.

### 2.5. Flow Cytometry Analysis of Immune Cells

#### 2.5.1. Analysis of Immune Cells in Blood

Flow cytometry was used to detect changes in immune cells in blood samples from each group of mice at 28, 42, and 56 Days after the first immunization.

Firstly, 1 µL of each of the following flow cytometry antibodies was added to a 1.5 mL EP tube containing 100 µL of mouse peripheral anticoagulated blood (with EDTA·2K anticoagulant): anti-mouse CD45 monoclonal antibody (mAb) 30-F11 (Super Bright™ 600, eBioscience™, eBioscience, Inc. (San Diego, CA, USA)), CD3 (BD Pharmingen™ FITC hamster anti-mouse CD3e. BD Biosciences (San Jose, CA, USA)), anti-CD4 mAb GK1.5 (eFluor™ 450, eBioscience™), anti-CD8a mAb 53-6.7 (PerCP-Cyanine5.5, eBioscience™), anti-CD44 mAb IM7 (APC, eBioscience™), and anti-CD62L (L-Selectin) mAb MEL-14 (PE, eBioscience™). The mixture was incubated at 4 °C in the dark for 30 min. Then, 1 mL of 1× RBC lysis buffer (prepared with deionized water) was added to the reaction system. After lysis at room temperature for 5 min, the mixture was centrifuged at 1500 rpm for 5 min at 4 °C, and the supernatant was removed to obtain the cell pellet. Subsequently, 200 µL of PBS (with 0.5% BSA) was added to the cell pellet, mixed by pipetting, and then centrifuged at 1500 rpm for 5 min at 4 °C to remove the supernatant. This washing process was repeated twice. Finally, the washed cells were resuspended in 500 µL of PBS (with 0.5% BSA), fixed with a final volume of 4% paraformaldehyde, and stored in the dark at 4 °C for subsequent detection.

At the same time, the same treatment was performed according to the following staining scheme: 1 µL of each of the following flow cytometry antibodies was added to tube 2: anti-CD45 mAB 30-F11 (Super Bright™ 600, eBioscience™), CD3 (BD Pharmingen™ FITC hamster anti-mouse CD3e), anti-CD4 mAb GK1.5 (eFluor™ 450, eBioscience™), anti-CD 69 mAb H1.2F3 (PE, eBioscience™), and CD103 (CD103 (Integrin alpha E) Monoclonal Antibody (2E7), APC, eBioscience™); 1 µL of each of the following flow cytometry antibodies was added to tube 3: anti-mouse CD19 (CD19 BUV395), IgM (IgM APC), and IgD (IgD FITC).

#### 2.5.2. The Analysis of Immune Cells in Splenic Single-Cell Suspension

After the mice were euthanized, and their spleens were placed on a 70 µm cell strainer, which was then placed in a 6 cm dish, 3 mL of PBS (with 0.5% BSA) was added, and the spleens were ground directly on the 70 µm cell strainer using the plunger of a 2 mL syringe. After grinding, the cell strainer was rinsed with 2 mL of PBS (with 0.5% BSA). Then, the splenic cell suspension was transferred to a 15 mL centrifuge tube and centrifuged at 1500 rpm for 5 min at 4 °C before the supernatant was removed. A 3 mL 1× RBC lysis buffer was added, and the mixture was vortexed and mixed, lysed at room temperature for 5 min, and then centrifuged at 1500 rpm for 5 min at 4 °C. After removing the supernatant, 3 mL of PBS (with 0.5% BSA) was added, vortexed, and mixed, and the 70 µm cell strainer was placed on a 50 mL centrifuge tube to filter the splenic cells again. The filtered cells were collected in a 50 mL centrifuge tube, centrifuged at 1500 rpm for 5 min at 4 °C, and the supernatant was removed. The cells were washed once with PBS containing BSA and then once with PBS without BSA. A total of 1 mL of PBS (with 0.5% BSA) was then added to resuspend the cells, which were vortexed and mixed, and connective tissue was removed with a pipette. The preparation of the splenic single-cell suspension was then completed. The splenic cells were suspended with 50 mL of PBS containing 0.5% BSA. The mixture was incubated at 4 °C for 10 min, and surface marker antibody incubation started without washing. Finally, staining was performed according to the color scheme in Section 2.5.1, and the mixture was incubated in the dark on ice for 30 min. After washing twice, 200 µL of 4% paraformaldehyde was added to fix the solution at room temperature in the dark for 30 min. The cells were then washed once and resuspended to 200 µL for subsequent detection.

#### 2.5.3. Gating Strategy

T cells were defined as the CD3^+^/CD45^+^ T cell population. Based on the differential expression of CD4 and CD8, T cells can be further divided into four major subpopulations: CD4^+^ Th cells, CD8^+^ Tc cells, CD4^+^/CD8^+^ (DP) T cells, and CD4^−^/CD8^−^ (DN) T cells. The latter are mainly composed of γδ+ cells under normal conditions. When analyzing CD44 and CD62L in TH and TC lymphocytes, three different subpopulations can be distinguished: CD62LhiCD44neg/lo naïve T cells, CD62LhiCD44hi central memory TCM cells, and CD62Lneg/loCD44hi effector memory TEM cells.

B cells were gated as the CD19^+^ cell population. When analyzing IgM and IgD in B cells, four different subpopulations can be distinguished: class-switched or activated B cells (IgM^−^IgD^−^), transitional B cells (IgM^+^IgD^−^), marginal zone B cells (IgM^+^IgD^+^), and naïve follicular B cells (IgM-IgD^+^).

### 2.6. Statistical Analysis

Statistical analysis was performed using GraphPad Prism 7.0 software. The data from each group were expressed as the mean ± SD of triplicates. Differences among the groups were analyzed using two-way ANOVA for multiple comparisons. A *p*-value of less than 234 was considered statistically significant.

## 3. Results

### 3.1. Weight Changes

**Figure 1:**
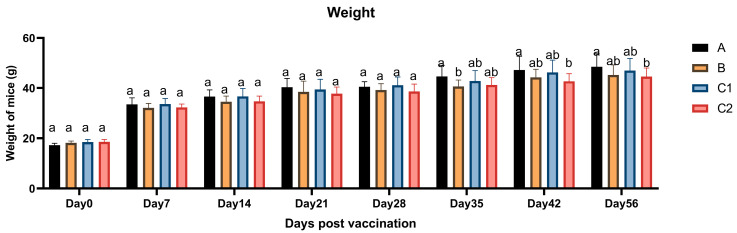
Changes in the body weight of experimental mice. The bar chart illustrates the body weight of the mice in each experimental group on Days 0, 7, 14, 21, 28, 35, 42, and 56 post-vaccination. The different letters over the data bars indicate whether their differences are statistically significant (*p* < 0.05) and vice versa (*p* > 0.05). If a group shows partial significant and non-significant differences with the preceding and following groups simultaneously, it is labeled with a combined letter (e.g., ab). This indicates that the group has no significant difference with at least one of the preceding (Group A) or following (Group B) groups, but there is a significant difference between the preceding and following groups themselves. The following figures are the same.

Figure 1 displays the body weight measurements of the mice in Groups A, B, C1, and C2 following vaccination. The average body weights across all groups showed no statistically significant differences (*p* > 0.05) at any of the eight time points, with all groups demonstrating a gradually increasing trend over time. These findings indicate that the vaccine adjuvants did not induce significant alterations in body weight, thereby confirming the biological safety of the immunological adjuvants.

### 3.2. Neutralizing/Antigen-Specific Antibody Levels

**Figure 2:**
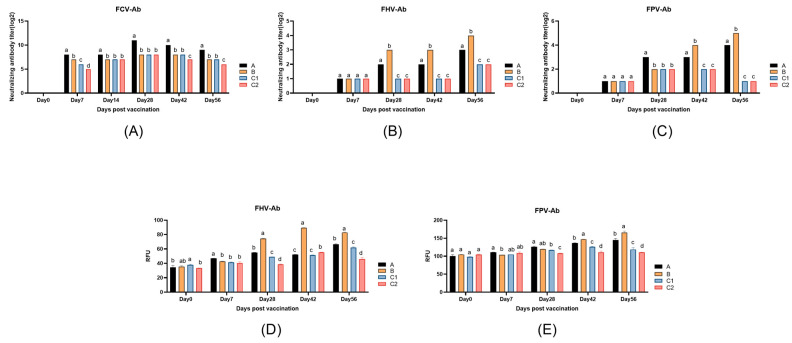
The changes in neutralizing antibody and specific antibody levels in the peripheral blood of experimental mice. (**A**) The neutralizing antibody titers against FCV (FCV-Ab); (**B**) the neutralizing antibody titers against FHV (anti-FHV Ab); (**C**) the neutralizing antibody titers against FPV (anti-FPV Ab); (**D**) the specific feline herpesvirus antibody (FHV-Ab); and (**E**) the specific feline panleukopenia virus antibody (FPV-Ab). The explanations of difference letters above the bar graphs is shown in Figure 1.

Figure 2A presents neutralizing antibody titers against FCV in Groups A, B, C1, and C2 on Days 0, 7, 14, 28, 42, and 56 post-immunization. On Day 7 post-primary immunization, Groups A and B showed significantly higher anti-FCV neutralizing antibody titers compared to Group C1 (*p* < 0.05). On Day 14, Group A maintained stable antibody levels and was significantly higher than the other three groups. Following booster immunization (Day 21), Group A demonstrated significantly higher anti-FCV neutralizing antibody levels than the other three groups at all subsequent time points (*p* < 0.05).

Figure 2B,C indicate that the neutralizing Ab titers against FHV-1 and FPV increased in Group A and B compared to Group C1 and C2 (*p* < 0.05), and the neutralizing Abs level of Group B was remarkably higher than that of Group A from Days 28 to Days 56 except for FPV on Day 28 (*p* < 0.05). There were no evident differences between the anti-FHV-1 and -FPV neutralizing antibodies of Groups C1 and C2 (*p* > 0.05).

Figure 2D and Figure 2E display the anti-FHV and FPV-specific antibody levels, respectively, measured on Days 0, 7, 28, 42, and 56. As shown in Figure 2D, Group A maintained higher anti-FHV-specific antibody levels than Group C2 on Days 28, 42, and 56 post-primary immunization (*p* < 0.05), while Group B showed significantly elevated anti-FHV-specific antibody levels compared to all other groups (*p* < 0.05). Both Groups A and B exhibited marked increases in specific antibody levels following secondary immunization (Day 21). Figure 2E reveals that Groups A and B displayed progressively increasing anti-FPV-specific antibody levels, with significantly higher titers than Groups C1 and C2 on Days 42 and 56 (*p* < 0.05). Notably, by Day 56, both Groups A and B achieved substantially enhanced anti-FPV-specific antibody levels compared to pre-booster measurements.

### 3.3. Blood Cell Analysis

**Figure 3:**
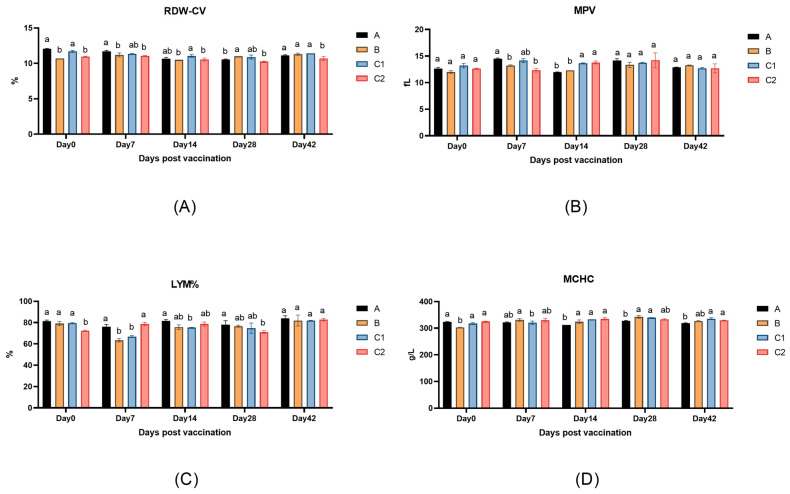
Changes in routine blood parameters in the peripheral blood of experimental mice. (**A**) The changes in red cell distribution width coefficients of variation (RDW-CV); (**B**) mean platelet volume (MPV); (**C**) lymphocyte percentage (LYM%); and (**D**) mean corpuscular hemoglobin concentration (MCHC). The explanations of difference letters above the bar graphs is shown in Figure 1.

Figure 3A–D present the peripheral blood parameters of mice across measurement time points, including the red cell distribution width (RDW-CV), mean platelet volume (MPV), lymphocyte percentage (LYM%), and mean corpuscular hemoglobin concentration (MCHC). The data demonstrate no statistically significant differences (*p* > 0.05) in these hematological indices among Groups A, B, C1, and C2 at any time point (Days 0, 7, 14, 28, 42, and 56), indicating that the adjuvants did not significantly affect murine hematological parameters.

### 3.4. Flow Cytometry of Immune Cells

**Figure 4:**
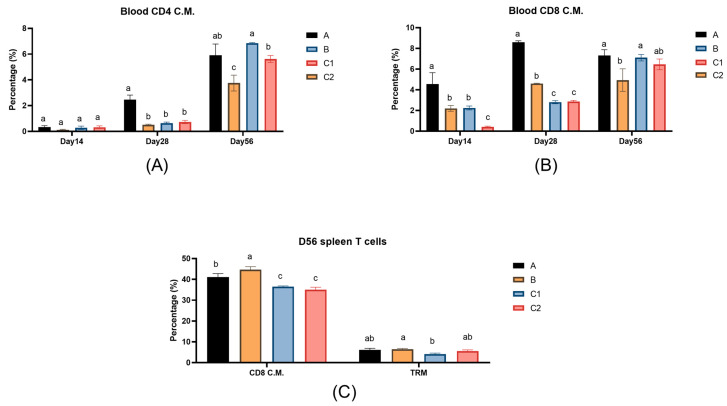
The percentage of T cells in the peripheral blood and splenocytes of experimental mice. (**A**) The bar graphs show the percentage of peripheral blood CD4 central memory T cells (TCM); (**B**) CD8 central memory T cells (TCM); and (**C**) the percentages of CD8 CMT cells and tissue-resident memory T cells (TRM) in the spleen of experimental mice on Day 56. The explanations of difference letters above the bar graphs is shown in Figure 1.

Figure 4A,B show the percentages of CD4 effector memory T lymphocytes and CD8 effector memory T lymphocytes in the peripheral blood of mice in Groups A, B, C1, and C2 on Days 14, 28, and 56 post-first immunization. Figure 4C shows the percentage of T lymphocyte subset with immunity in the spleens of mice in Groups A, B, C1, and C2 on Day 56. In the blood T cell analysis on Day 28, Group A demonstrated significantly higher percentages of both CD4^+^ and CD8^+^ central memory T cells (TCMs) compared to the other three groups (*p* < 0.05), while no significant differences were observed for Group C2 by Day 56 (*p* > 0.05). For the splenic T cell analysis on Day 56, the percentages of CD8 central memory T cells in Groups A and B were significantly higher than those in Groups C1 and C2 (*p* < 0.05), while there was no significant difference in tissue-resident memory T cell levels between Groups A/B and Group C2 (*p* > 0.05).

**Figure 5:**
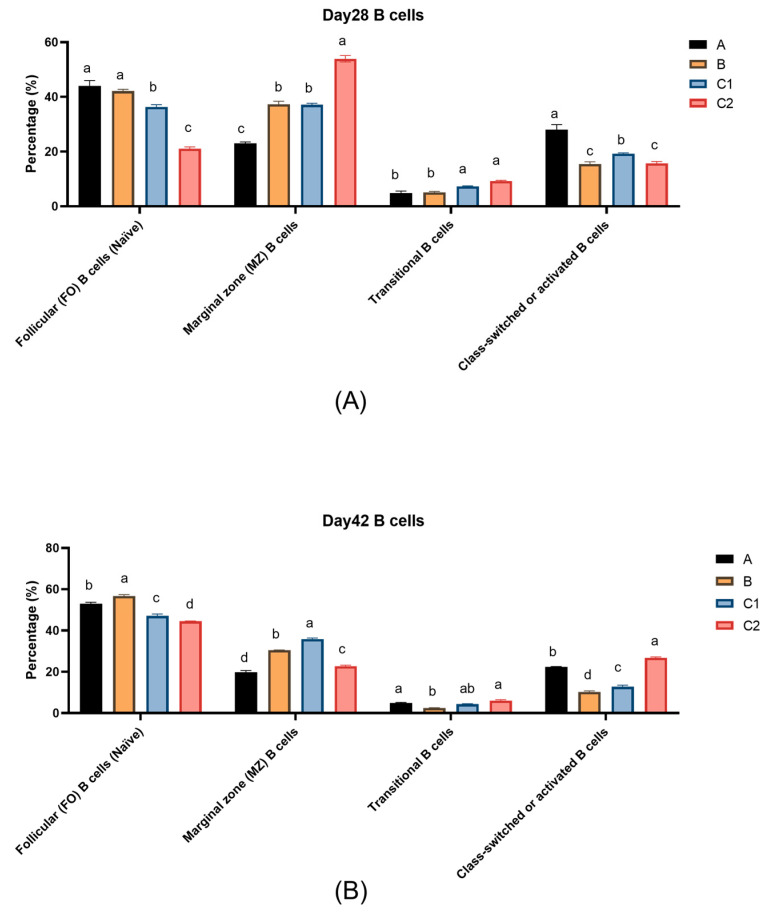
Changes in the percentage of B cell subsets in the peripheral blood of experimental mice on Day 28 (**A**) and Day 42 (**B**). The explanations of difference letters above the bar graphs is shown in Figure 1.

Figure 5A,B show the percentages of B lymphocyte subsets in the peripheral blood of mice in Groups A, B, C1, and C2 on Day 28 and Day 42. At both measurement time points, transitional B cells (IgM^+^IgD^−^) were present in low amounts in all groups. The follicular B cells (IgM^−^IgD^+^) and activated B cells (IgM^−^IgD^−^) in Groups A and B were significantly higher than those in Groups C1 and C2 (*p* < 0.05). Moreover, the level of transitional B cells (IgM^+^IgD^−^) on Day 56 decreased compared to Day 28. On Day 28, the activated B cell level in Group A was significantly higher than that in the other three groups (*p* < 0.05).

## 4. Discussion

In recent decades, commercial vaccines for cats have exhibited suboptimal efficacy [25,26]. Vaccines against FCV and FHV infections may show waning immunity over time, necessitating periodic booster vaccinations to sustain protective effects [27]. Conversely, immune adjuvants can augment vaccine immunogenicity and durability by prolonging antigen retention and fostering immune cell activation. To date, no studies have explored Mn, cytokines, or metabolic modulators as adjuvants in feline vaccines. Although aluminum adjuvants have proven moderately effective and are widely used, they are unable to elicit robust cellular immune responses and may induce localized inflammatory reactions [28], rendering them unsuitable for feline vaccine formulations. Mn possesses stable chemical properties and can establish an immunostimulatory microenvironment [10]. In a rabies vaccine study, manganese jelly (MnJ) significantly increased the production of various immune cells [29]. However, the immunomodulatory effects of Mn are constrained by specific immune microenvironments [30]. Furthermore, improper dosage control may lead to gradual Mn accumulation in the body upon repeated administration, potentially causing neurotoxicity and reproductive toxicity.

In contrast, cytokines and metabolic regulatory molecules offer significant advantages as novel immune adjuvants. Cytokines can enhance immune responses while mitigating adverse effects [31,32]. In this study, IL-15 and IL-23 were selected as two representative cytokines with crucial immunomodulatory functions. Research indicates that IL-15 regulates the generation and maintenance of memory T cells, enhances cytotoxic activity, and promotes cytokine production [33]. Given its role in memory T cell development and persistence, IL-15 has been incorporated as a vaccine adjuvant for infectious diseases [34]. IL-23 can amplify immune potency by promoting the expansion of tissue-resident memory T (TRM) cells [35,36]. Moreover, in vitro studies have shown that the combined use of IL-15 and IL-23 can effectively facilitate the generation of CD8^+^ central memory T cells [37].

Metabolic regulatory molecules, which are endogenous compounds, can also enhance immune efficacy while reducing adverse reactions such as inflammatory responses and oxidative stress. Nicotinamide adenine dinucleotide (NAD) and its precursor β-nicotinamide mononucleotide (NMN) enhance macrophage phagocytic capacity and suppress inflammatory reactions [16,21]. N-acetylcysteine (NAC), one of the most extensively studied antioxidant agents [18], modulates immune function by regulating T cell subsets and upregulating the ability of dendritic cells to activate T cells, thereby increasing CD8^+^ T cell frequency and promoting memory T cell expansion [20]. L-carnitine enhances immune efficacy by boosting immune cell activity and attenuating inflammatory responses [19]. Additionally, sodium butyrate and serine can substantially influence immunity by modulating immune cell function and the production of immunologically active substances [15,17].

This study evaluated the biosafety of immune adjuvants by examining body weight and hematological parameters in experimental mice. The results revealed no significant differences in body weight among the groups at any measured time point, with all groups exhibiting a stable upward trend in weight gain. This suggests that the adjuvants did not exert any adverse effects on the growth and development of the mice. Furthermore, no significant differences were observed in peripheral blood hematological parameters across the four groups, indicating that IL-15, IL-23, and related metabolic molecules did not negatively impact the hematopoietic system or blood composition, further confirming the biosafety of the adjuvants.

CD4^+^ and CD8^+^ T cell memory play a pivotal role in combating infectious diseases. Upon encountering foreign antigens, naïve CD8^+^ T cells proliferate and differentiate into effector CD8^+^ T cells, subsequently leaving behind a stable population of CD8^+^ memory T cells after infection resolution [38]. CD4^+^ T cells indirectly participate in immune responses by modulating the activity of other immune cells, whereas CD8^+^ T cells directly mediate cytotoxic effects to eliminate virus-infected cells. Central memory T cells (TCMs) can persist long-term in the body and mount rapid responses upon re-exposure to the same antigen, thereby providing long-term immunity. The results demonstrated that Group A exhibited a significant increase in blood T cell levels after secondary immunization, with no significant difference compared to Group C2 on Day 56, and also remained significantly higher than Group B. This indicates that Group A enhanced the immune response in mice post-booster immunization while maintaining a protective efficacy comparable to that of commercially available vaccines. On Day 56, the percentages of splenic CD8^+^ TCM cells in Groups A and B were significantly higher than those in Groups C1 and C2. These findings suggest that IL-15 and IL-23 promote the expansion of tissue-resident memory T cells (TRMs) and TCM, while metabolic molecules contribute to elevated CD8^+^ T cell levels and facilitate the formation of memory T cells.

For B cell subsets, upon activation, follicular (FO) B cells enter the germinal center and ultimately differentiate into plasma cells or memory B cells [39]. Activated B cells signify the maturation of the immune response, and a subset of them further differentiate into memory B cells, which persist long-term in the body and mount rapid responses during secondary immune reactions [40]. Appropriate NAD^+^ levels help maintain normal B cell function, promoting their differentiation into plasma cells for antibody production. Meanwhile, NMN supplementation may enhance B cell immune responses by elevating NAD^+^ levels [40]. Experimental data demonstrate that Groups A and B, supplemented with metabolic regulators and cytokines, exhibited significant B cell differentiation characteristics: a reduced proportion of transitional B cells and a notably higher proportion of FO B cells compared to the control group. The addition of cytokines IL-15/23 may have facilitated the differentiation of transitional B cells into FO B cells, laying the foundation for the immune system to produce more high-affinity antibodies against antigens. The percentage of marginal zone (MZ) B cells in Groups A and B was lower than in Group C1, suggesting that the adjuvants in Groups A and B may have activated MZ B cells, driving their differentiation into plasma cells or other subsets [41]. Furthermore, Group A displayed the highest percentage of activated B cells on Day 28, indicating that more activated B cells can subsequently differentiate into plasma cells and memory B cells, thereby enhancing antibody production and long-term immunity.

The neutralizing antibody titer is closely associated with vaccine protective efficacy. The level of neutralizing antibodies in Group A was significantly higher than those in other groups after booster immunization, indicating that the adjuvants IL-15/23 significantly enhanced the immune response. The results of anti-FHV and anti-FPV neutralizing antibodies do not have good consistency with their IFAT results due to systematic method differences and analysis deviation. The FHV-Ab measurement results demonstrate that the specific antibodies in Group B increased significantly after booster immunization and exhibited an upward trend. On Day 56, the FHV-Ab level in Group B was significantly higher than those in the other three groups, suggesting that the cellular metabolic molecules could markedly and persistently enhance immune efficacy. After Day 28, the anti-FPV-specific antibody levels in Groups A and B were consistently higher than those in Groups C1 and C2, indicating that these metabolic regulators could more effectively promote the production of FPV-specific antibodies post-booster immunization. These results demonstrate that the combination of metabolic modulators and cytokines as adjuvants can optimize B-cell immune responses through multiple pathways, significantly improving antibody production efficiency and establishing long-term immune memory.

## 5. Conclusions

Our study demonstrates how feline IL-15, feline IL-23, and a series of metabolic regulatory molecules, when used as immunological adjuvants for the feline trivalent vaccine, exhibit a certain level of safety. During the immune response process, these adjuvants effectively promote the generation and differentiation of immune cells while also enhancing the production of antibodies. This finding indicates that the aforementioned composite adjuvant can serve as an effective immune enhancer for the feline trivalent vaccine, providing a new potential strategy and theoretical basis for optimizing the immunological efficacy of the feline trivalent vaccine.

## Figures and Tables

**Table 1:** Experimental mouse immunization grouping.

Group	Treatment
A	0.1 mL of antigen + feline IL-15 (0.5 µg per mouse) + feline IL-23 (0.5 µg per mouse) + NMN (50 µg per mouse) + NAD (50 µg per mouse) + N-Ace (0.6 mg per mouse) + sodium butyrate (1.2 mg per mouse) + serine (1.2 mg per mouse) + L-carnitine (1.2 mg per mouse)
B	0.1 mL of antigen + NMN (50 µg per mouse) + NAD (50 µg per mouse) + N-Ace (0.6 mg per mouse) + sodium butyrate (1.2 mg per mouse) + serine (1.2 mg per mouse) + L-carnitine (1.2 mg per mouse) + Mn (200 µg per mouse)
C1	0.1 mL of antigen + Mn (200 µg per mouse)
C2	0.1 mL of commercial vaccine + 0.1 mL of PBS

Abbreviations: IL: interleukin; NMN: nicotinamide mononucleotide; NAD: nicotinamide adenine dinucleotide; N-Ace: N-acetylcysteine; PBS: phosphate-buffered saline.

## Data Availability

The datasets generated or analyzed during the current study are not publicly available due to commercial confidentiality but are available from the corresponding author on reasonable request.
